# FREQUENCY OF WRIST GROWTH PLATE INJURY IN YOUNG GYMNASTS AT A TRAINING CENTER

**DOI:** 10.1590/1413-785220162404157422

**Published:** 2016

**Authors:** María Roxana Viamont Guerra, Jose Renato Depari Estelles, Yussef Ali Abdouni, Diego Figueira Falcochio, Joao Roberto Polydoro Rosa, Liane Hulle Catani

**Affiliations:** 1. Irmandade da Santa Casa de Misericórdia de São Paulo, Department of Orthopedics and Traumatology, São Paulo, SP, Brazil.; 2. Irmandade da Santa Casa de Misericórdia de São Paulo, Department of Pediatrics, São Paulo, SP, Brazil.

**Keywords:** Athletic Injuries, Child, Adolescent, Wrist

## Abstract

**Objective:**

To assess the frequency of physeal injuries and wrist pain in young competitive gymnasts according to their training characteristics***.***

**Methods:**

This is a cross-sectional study (January-June 2015) of a male gymnastics team in São Paulo, SP, Brazil. Nineteen gymnasts, mean age 13.3 years, were evaluated in three ways: a questionnaire, physical examination and radiographs***.***

**Results:**

On average, they trained since 6 years-old and during hours per week. Eighty-two percent had wrist pain and 65% had wrist physeal injury. The pain was worse in practitioners of (82%) and soil (17%) exercises. A greater frequency of physeal injury was found in those with more years of training and higher weekly working hours, wrist pain was more frequent in those with higher weekly working hours, and a decreased range of motion was observed in those with physeal injury, results statistically significant***.***

**Conclusions:**

We found that 65% of gymnasts had wrist physeal injury and 82% had wrist pain. There were statistically significant relationships between physeal injury and years of training, physeal injury and weekly working hours, pain and weekly working hours, and physeal injury and range of motion. ***Level of Evidence IV, Case Series.***

## INTRODUCTION

The presence of children and adolescents in competitive sports practice has become increasingly frequent. Artistic gymnastics (also known as Olympic gymnastics) is one of the sports where specialization occurs at earlier ages, with intense training to achieve high performance and reach elite levels. Thus, the risk of injury in this population, including specific conditions of the immature skeleton, has become concern of federations, parents, coaches and physicians, requiring greater care in the multidisciplinary approach of these young athletes.

The types of injuries vary according to gender^1^, type of sports modality, training intensity,^2^ age and age of training start,^3^ among others. Injuries can be acute (macrotrauma) or chronic (microtrauma), the latter being strongly associated to overloading and overuse.

The longest duration and intensity of training can lead to overload in the immature skeleton. Chronic wrist pain in young gymnasts is a good example of these conditions, also observed in the distal femur of runners and proximal humerus of baseball pitchers.^4^

The learning curve and gymnast training requires the use of strenuous repetitive movements, which often use the wrist as a loading zone, and forces far exceed their body weight. The wrist is often in dorsiflexion with the stress applied to ulnar and radial deviation movements.

The prevalence of wrist pain in young gymnasts, according to the literature, ranges from 32 to 79%, and 56-67% in best quality studies.^5^ ^-^ ^8^ Since the 80's a relationship between pain and radiographic alterations in young gymnasts' wrist has been observed.^9^ They had mostly pain in the dorsal region of the wrist and the radiographic findings included enlargement of the distal radius growth plate, metaphyseal bone cysts, distal wedging of the epiphysis and blurring of the radiolucent physis area.^9^

At that time, Roy et al.^9^ questioned what would be the future outcome of these changes. Currently, the development of long term positive ulnar variance and bone bar formation in the distal radius physis has been observed, associated with evidence of physeal injury.^10^

Information about the consequences of wrist injuries related to sports practice in young athletes is still limited, as well as its relationship to pain. These children and adolescents often continue their training even with the complaint of pain, risking future serious implications.

Despite the sports gesture is similar for all athletes, training rhythm, guidance, and monitoring of these young athletes vary from one population to another.

The objective of this study was to evaluate the frequency of physeal injuries and pain in competitive young gymnasts' wrists and some characteristics of their training in a Brazilian gymnastics training center.

## MATERIALS AND METHODS

A cross-sectional study was conducted to assess the presence of pain and injury of the physis of gymnasts' wrists from a male team at a gymnastics training center in São Paulo, SP, Brazil, between January and June 2015. The study included 19 gymnasts, aged between 9 and 18 years (mean 13.3 years old). Both wrists were evaluated, regardless of pain complaints.

Inclusion criteria were practicing competitive sport at national or international level, and the presence of open physis on radiographs. The study excluded athletes who had traumatic injuries associated with upper limb and those who did not have X-rays.

The evaluation of the gymnasts was made in three stages. First, a questionnaire was applied to athletes, which included the following items: age of onset of sports practice; years of training; number of hours of weekly training; performed modalities and exercises; presence of wrist pain, duration and intensity (by visual analogue pain scale); restrictions on training due to pain and if there was any reduction in performance.

In the second stage, a physical examination took place, where the following parameters were assessed: pain to palpation, active and passive arc movement including flexion, Extension, radial deviation and ulnar deviation (measured with a goniometer).

As a third step of the study, we evaluated the bilateral, anteroposterior and lateral views, and absolute profile of wrists radiographs, in order to evaluate the appearance of the distal physis of the radius and ulna. We considered as signs of injury on the growth plate: enlargement of the distal radial physis, metaphyseal bone cysts, wedging distal epiphysis and blurring of the radiolucent physis area.^11^ The inclination ulnar and volar angle of the radius were also measured.

These data were, then, statistically analyzed by Mann-Whitney test and chi-square test using SPSS V17 software, Minitab 16 and Office Excel 2010. Statistical significance was considered at *p*<0.05.

All study participants or their legal guardians signed a Free and Informed Consent form. The Ethics Committee approval number is CAAE 44941715.5.0000.5479.

## RESULTS

The evaluated gymnasts started their training between 4 and 9 years of age (mean age 6 years old), practicing on average for 7.8 years, training on average 25.7 hours weekly.

The training was conducted continuously in all units of the male artistic gymnastics (floor, pommel horse, jumping table, parallel bars, horizontal bar and rings). All athletes had national and/or international competitive levels.

The prevalence of wrist pain was 82%, 53% bilaterally. Of those who had wrist pain, the average time for the manifestations of pain was 10.9 months and the intensity of pain (VAS) averaged 3.7 points.

Among those who complained of wrist pain, 47% reported that the pain limited their performance during training. The pain was reported during specific modalities in training: horse (82%), soil (17%) and parallel bars (12%). Only one athlete had to interrupt training for 21 days. On physical examination, the range of motion amplitude is shown in Table 1.

**Table 1:** Mean values of active and passive arc of movement of the wrists.

	Active Flexion	Passive Flexion	Active Extension	Passive Extension	Active radial deviation	Passive radial deviation	Active ulnar deviation	Passive ulnar deviation
Right wrist	66º	86.9º	59.1º	76.7º	22.3º	34.1º	35.6º	48.7º
Left wrist	68.5º	85.8º	57.3º	74º	26.2º	36.8º	34.3º	48.9º

**Figure 1:**
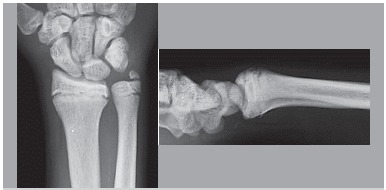
Front and profile x-ray of the right wrist of a young athlete with complain of wrist pain. Observe the enlargement of the distal physis of the radius and ulna and blurring of the radiolucent physis area.^11^

**Figure 2:**
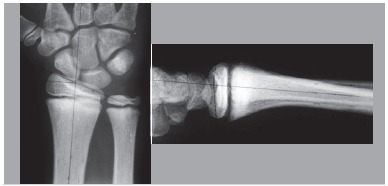
Front and profile x-ray of the left wrist of a young athlete without complain of wrist pain Observe the marks at ulnar (20º) and radial (9º) inclinations, respectively.

Physis injuries were found in radiographs in 65% of athletes, 53% bilaterally. (Figure 1) Furthermore, in the anteroposterior radiographs of the wrist, the average value of radio ulnar inclination was 18.1º, and in the wrist profile radiograph, the average volar inclination of the radius was 6.7º. (Figure 2)

**Figure 3:**
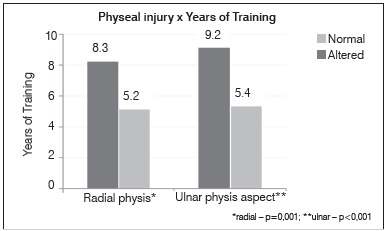
Relationship between radial and ulnar distal physeal injury and numbers of training years.

There was no difference in the association between physeal alterations with the age of onset of sports practice. However, there was a statistically significant difference in physis injury when the following variables were taken into account: years of training and hours of training per week. In these cases, the means were always higher in the group with radiographic change (*p*<0.05). (Figures 3 and ^4^)

**Figure 4:**
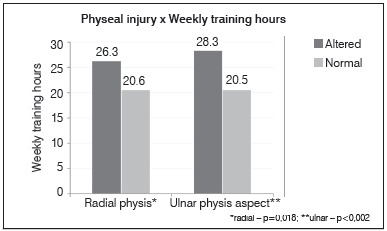
Relationship between radial and ulnar distal physeal injury and weekly training hours.

Regarding wrist pain, there was only a statistically significant difference regarding the weekly working hours, so that those who had wrist pain trained for more hours per week (*p*=0.037). As for the relationship between wrist pain and years of training or age of onset of sports practice, there was no statistically significant difference.

Assessing the range of motion of the wrists, we observed a statistically significant decrease in passive Extension and active and passive radial deviation in cases where the appearance of the radial physis was altered (*p*<0.05, Table 2).

**Table 2:** Relationship between radial physis aspect and arc of movement.

Physis aspect	Radius Distal	Mean	Standard deviation	N	IC	*p*-value
Active flexion	Altered	67.2	9.8	39	3.1	0.187
	Normal	72.4	12.1	18	5.6	
Passive flexion	Altered	85.4	13.1	39	4.1	0.103
	Normal	90.8	11.8	18	5.5	
Active extension	Altered	59.4	9.2	40	2.9	0.103
	Normal	62.8	7.8	18	3.6	
Passive extension	Altered	73.8	9.2	39	2.9	<0.001
	Normal	85.3	7.1	18	3.3	
Active radial deviation	Altered	22.5	5.6	35	1.9	<0.001
	Normal	28.7	5.4	18	2.5	
Passive radial deviation	Altered	33.3	6.8	35	2.3	<0.001
	Normal	43.0	5.7	18	2.6	
Active ulnar deviation	Altered	36.5	5.2	35	1.7	0.566
	Normal	35.5	5.4	18	2.5	
Passive ulnar deviation	Altered	49.8	5.1	35	1.7	0.831
	Normal	49.7	7.8	18	3.6	

Regarding the analysis of the ulnar and volar inclination angles radio on x-rays, it was found that those with wrist pain had a higher average ulnar inclination of the radius (19.1º) than those who had no pain (16.2º), with *p*=0.021. We found a trend for increased volar inclination of the radius in patients reporting wrist pain, but without statistical significance (7.8º in gymnasts with pain, 4.3° in those without pain, *p*=0.067). There was no relationship between distal physeal injury of the radius and wrist pain (*p*=0.493) and distal physeal injury of the ulna and wrist pain (*p*=0.455), which shows them to be independent variables. 

## DISCUSSION

The increasing competitiveness and technical level in gymnastics are taking every time younger children to intense training routine aiming performance and high yield. Much has been discussed in the literature on the implications of these activities in the immature skeleton. Overload on the wrist of these athletes can become a threatening factor for their career, and can lead to harmful future consequences.

Studies show that the age of onset of this sport ranges from 3 to 12 years old, an approximate average of 5 and 7 years old,^7^ ^,^ ^12^ ^,^ ^13^ which is in line with the mean age found in this study, of 6 years old on average (range, 4 to 9 years). There were no studies mentioning the time elapsed since the start of training.

As for the training intensity, in terms of weekly working hours, the literature shows that elite gymnasts often train on average 27-36h per week,^9^ ^,^ ^12^ ^,^ ^13^ higher than average in this study (25.7h per week).

In a recent systematic review, Kox et al.,^3^ in 2015, observed a prevalence of wrist pain in young athletes of 32-73%, narrowing to 56-67% in best quality studies.^7^ ^.^ ^8^ Very high numbers that can reach 82%, as in the present study. The correlation of pain with clinical findings and possible outcomes remains unclear. 

DiFiori et al.^7^ observed in a study with 59 gymnasts that the most important factors associated with wrist pain were high technical level, older athletes with more years of training, while in this study it was found that there are statistically significant only for wrist pain and increased weekly working hours. In the same study, as in others, an association between wrist pain and radiographs with physeal injury has been described.^5^ ^-^ ^9^ ^,^ ^14^ ^-^ ^16^ Unlike the present study, in which there was no significant association between injury in the distal physis of the radius or ulna and wrist pain.

The subjectivity of pain perception may be an obstacle when it comes to a pediatric athletic population. Nemeth et al.^11^ observed in a group of 68 Olympic level patients aged 6-13 years old, that the older ones (over 11 years old) have a better characterization of pain and understanding of its implications than the younger ones, mostly 6-8 year old. The authors warned about the risks that many of them understand pain as something to be overcome to improve performance, as if it were part of the training. In the present study, it was observed that, although there was wrist pain complaints in 82% of gymnasts, its intensity (according to the visual analog pain scale) showed an average of 3.7 points, a reason probably most athletes did not miss trainings. However, 47% of gymnasts reported that the pain limited their performance.

The sports modalities that worsened pain were the pommel horse (82%), soil exercises (17%) and parallel bars (12%), probably because those are activities that increase the radio-carpal impact. DiFiori et al.^6^ ^,^ ^7^ also showed that the activities that were associated with wrist pain were soil exercises and pommel horse.^14^

During the physical examination, it was found that only five gymnasts had pain reproduced by palpation, similar to that observed by DiFiori et al.,^7^ in which only two of the 33 athletes had it.

Some authors noted some advantages in gymnastics practice in the pediatric population. In a study with 84 girls training up to 16h per week, musculoskeletal benefits in the distal third of the forearm were observed in up to 66% of cases, such as improved strength and bone mass.^2^ In pre-pubertal children training at recreational and precompetitive levels, a better bone resistance was also observed.^17^ This data demonstrated that there can be a fine line between sports practice leading to benefit or injuries caused by abuse.

Radiographic changes in the young gymnast's wrists began to be observed in the 80s,^9^ ^,^ ^18^ ^,^ ^19^ such as blurring or enlargement of the physeal plate, cystic alterations, epiphysis wedging and possible bone bars. Although well described, they depend on well-trained professionals for assessment, and may sometimes go unnoticed. The advent of magnetic resonance imaging could have brought greater sensitivity and specificity.^19^ ^,^ ^20^

Regarding the association between physeal injury and number of weekly training hours or more training years, present in this study, another study found a significant relationship only for physeal injury and higher weekly workload, without significance for physeal injury and years of training.^7^

It was not found in the literature a comparison of radiographic changes of angular parameters and pain. In this study, we observed a significant increase in ulnar inclination (*p*=0.021) and a tendency to increased volar inclination of the radius (*p*=0.067) in patients with wrist pain. Probably, with a larger "n" we would be able to confirm this trend. It was also not find in the literature any relation between the limitation of the range of arc of movement of the wrist and radiographic changes, while in this study, we observed a significant reduction in passive Extension and active and passive radial deviation of physeal injured wrists.

A limitation of this study was the low sample obtained and difficulty to obtain the athletes' adherence to the protocol. However, even with limited data, some correlations between pain, radiographic abnormalities, and other clinical aspects have been observed, obtaining significant relationship between physis injury and years of training, physis injury and hours of training per week, pain and training hours a week, physeal injury and change in range of motion, and finally, pain and alteration of the ulnar inclination angle of the radius.

The clinical outcome of these changes is not yet well established, as well as the implications of injuries in the development of young athletes, however, with the identification of risk factors and specific effects, many injuries could be prevented or treated in due time. Federations, parents, coaches and physicians should be aware that young gymnasts are at high risk for developing abuse injuries that cause pain and skeletal changes, which can lead to a number of complications that can be limiting and threaten promising careers.

## CONCLUSION 

We found in this study that 65% of gymnasts had physeal injury in the wrist and 82% had wrist pain, but there was no relationship between these variables. It was also possible to observe that the frequency of physeal injury was higher in athletes who had more years of training and higher weekly working hours. Wrist pain was more common in those with higher weekly working hours, there was a decrease of arc of movement in those with physeal alterations and increased ulnar inclination angle of the radius in those with wrist pain.
